# Intraventricular Craniopharyngiomas—Overcoming Their Relative Inaccessibility: Institutional Experience With a Review of Literature

**DOI:** 10.3389/fneur.2021.755784

**Published:** 2021-11-15

**Authors:** Chandrashekhar Deopujari, Sanjay Behari, Krishna Shroff, Ashutosh Kumar, Bhushan Thombre, Vikram Karmarkar, Chandan Mohanty

**Affiliations:** ^1^Department of Neurosurgery, Bombay Hospital Institute of Medical Sciences, Mumbai, Maharashtra University of Health Sciences, Nashik, India; ^2^Department of Neurosurgery, Sanjay Gandhi Post Graduate Institute of Medical Sciences, Lucknow, India

**Keywords:** craniopharyngioma, intraventricular craniopharyngioma, intraventricular tumor, adult craniopharyngioma, hydrocephalus

## Abstract

**Introduction:** Craniopharyngiomas constitute 2–4% of intracranial neoplasms. Intraventricular craniopharyngiomas (IVCrs) are the rarely encountered varieties of these lesions.

**Objective:** The objective of the study was to study the special features in clinical presentation, imaging, management, and surgical outcome of IVCrs.

**Materials and Methods:** This retrospective analysis included the combined experience from two tertiary care institutions. Medical records of histopathologically proven cases of IVCrs from January 1994 to June 2021 were assessed, and images were analyzed based on the criteria by Migliore et al. for inclusion of solely intraventricular lesion with the third ventricular ependyma demarcating it from the suprasellar cistern.

**Results:** Among the 25 patients included (mean age: 35.4 years), the most common presentation included headache (*n* = 21, 84%), vomiting and other features of raised ICP (*n* = 18, 72%), visual complaints (*n* = 12, 48%), and endocrinopathies (*n* = 11, 44%). Fifteen had predominantly cystic tumors, two were purely solid, and eight were of mixed consistency. Primary open microsurgical procedures were performed in 18 (72%) patients, of which four (16%) were endoscope-assisted. Seven (28%) underwent a purely endoscopic procedure. One underwent a staged surgery with endoscopic cyst fenestration and intracystic interferon (IFN)-alpha therapy, followed by microsurgical excision. Complete excision was achieved in 10 patients, near-total in nine, and partial excision in six. Four patients underwent a ventriculoperitoneal shunt (one before the definitive procedure). At a median follow-up of 36 months (range:11–147 months), five patients developed a recurrence, and one had a stable small residue. This patient and two others with small cystic recurrences were observed. One patient was managed with radiotherapy alone. Another underwent re-surgery after a trial of radiotherapy, and the last patient developed a local recurrence, which was managed with radiotherapy; he then later developed an intraparenchymal recurrence, which was operated.

**Conclusion:** Purely IVCrs present with raised intracranial pressure, and visual disturbances are less common. Their deep-seated location and limited surgical field-of-view makes minimally invasive endoscopic-assisted surgery most suitable for their excision. The thin-walled cystic lesions may be occasionally adherent to the ependymal wall in close vicinity to the thalamus–hypothalamus complex, making complete excision difficult. Their responsiveness to radiotherapy, often leads to a gratifying long-term outcome.

## Introduction

Craniopharyngiomas are benign tumors, originating either from squamous epithelial cell that rests in the Rathke's pouch, located along its path from the nasopharynx to the infundibulum, or as a result of squamous metaplasia from the pars tuberalis of the pituitary gland (1–4). They constitute 2–4% of all intracranial tumors (3, 5–12). Multiple classification systems (based on their imaging features) have been proposed to help in choosing the appropriate management strategy (13). Purely intraventricular variety of craniopharyngiomas are infrequent, with the reported incidence varying from 0.5 to 14% of all craniopharyngiomas (3, 5, 8, 12, 14). As IVCrs originate from ectopic remnants present within the neuraxis, they are generally seen in older patients and present more often with features of raised intracranial pressure, rather than with visual or endocrinological disorders, as compared with their sellar/suprasellar or suprasellar–intraventricular counterparts (5, 11, 12, 15). Their deep-seated location, proximity, and often adherence to the walls and floor of the third ventricle and the hypothalamus, makes their surgical excision challenging. In this study, we discuss the clinical and radiological features, and management nuances of purely IVCrs.

## Methodology

### Patient Selection

**Figure 1:**
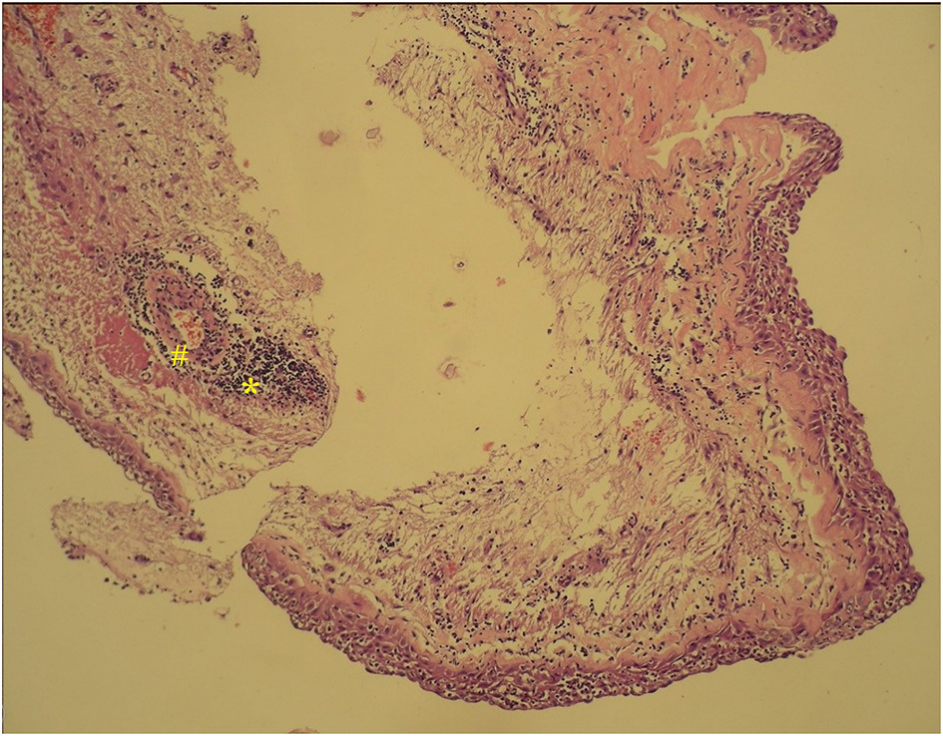
Histopathology section of an adamantinomatous intraventricular craniopharyngioma, showing areas of wet keratin (#) with calcification (*).

**Figure 2:**
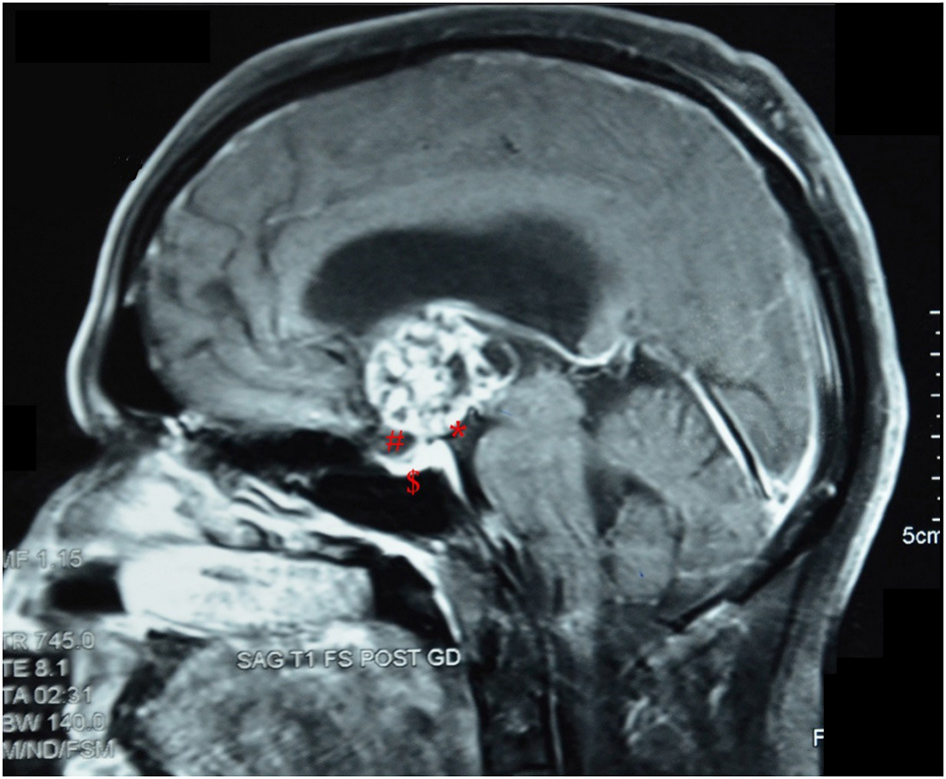
Post-contrast sagittal MRI image of a patient with an intrinsic third ventricular craniopharyngioma, showing features as described by Migliore et al. (16). *, an intact third ventricular floor; #, a patent suprasellar cistern; $, absence of sellar abnormalities.

The medical records of operated and histologically proven (Figure 1) cases of craniopharyngiomas, managed between January 1994 to June 2021 (The experience of the senior author from the first institution, and the departmental experience from the second institution is reported) were assessed. The purely intraventricular tumors were selected based on the radiological inclusion criteria suggested by Migliore et al. (16). This includes an intraventricular location of the tumor with the ventricular ependyma being intact inferiorly, clearly demarcating the tumor from the unoccupied suprasellar cistern (Figure 2). The demographic, clinical, endocrinological, and radiological features of these purely IVCrs were analyzed. The surgical approaches undertaken, their merits and demerits, and the subsequent adjuvant therapy administered, were assessed.

### Follow-Up

The follow-up data regarding visual/endocrine status, postoperative complications, postoperative radiotherapy administered, and the recurrence rate were also analyzed.

### Statistics

A descriptive analysis of the data obtained has been reported.

### Review of Literature

A search was made on the PubMed database using the keywords “intraventricular craniopharyngioma,” and the articles which were intended for the reporting of cases of purely intraventricular craniopharyngiomas were selected, and their findings are summarized in Table 1. The data of similar such case series were used for comparison with the results obtained from our series of patients.

**Table 1:** Prior reports and series of purely intraventricular craniopharyngiomas in literature.

**Reference**	**No. of cases**	**Age (years)**	**Sex**	**Clinical presentation**	**Preoperative visual status**	**Preoperative hormonal status**	**Imaging appearance**	**Extent of surgical excision**	**Outcome**	**Recurrence**
Cashion and Young (17)	2	46	Male	Headache, malaise, papilloedema	-	-	-		Died	-
		26	Male	Headache, lethargy, episodes of syncope, papilloedema	-	-	-	Underwent ventriculojugular shunt	Died	-
King (18)	4	68	Female	Gradual diminution of vision especially in left eye, early somnolence, decline in memory	Visual acuity-6/12 right eye and 6/18 left eye, bitemporal hemianopia, more severe in left eye	-	Pneumoencephalogram-third ventricular mass	Subtotal excision	Improved and remained well over following 6 years	No
		10	Male	Headaches, vomiting, papilloedema	Visual acuity of 6/12 in either eye with incomplete left homonymous hemianopia	Had diabetes insipidus, decreased sexual characteristics	Pneumoencephalogram-calcified tumor in third ventricle	Underwent ventriculocisternotomy followed by radiotherapy initially, on progression underwent subtotal excision	Developed memory deficits, poikilothermia, visual fields improved postoperatively	No
		54	Male	Progressive diminution of vision, intellectual dullness	Visual acuity of 6/9 in right eye and finger counting in left eye, visual fields showed bitempoal hemianopia, scotomatous on the left, fundi showed optic atrophy		Pneumoencephalogram-showed tumor in third ventricle	Subtotal excision	Euphoria, developed homonymous hemianopia, visual acuity-6/9 in right eye and finger counting in left eye, required ventriculo-atrial shunting due to persistent ventricular enlargement, underwent radiotherapy, hypopituitarism treated with supplementation	No
		47	Male	Headaches and loss of vision	Visual acuity of 6/60 in right eye and recognition of hand movements in left eye	On thyroxine supplementation	Calcified cystic intraventricular craniopharyngioma	Subtotal excision leaving behind a small piece attached to floor of the third ventricle just behind the optic chiasm	Postoperatively developed some confusion, serum hyperosmolarity requiring antidiuretics, tendency to poikilothermia, visual acuity 6/18 on right and 3/60 on left, also required steroid replacement	
Goldstein et al. (19)	1	57	Female	Severe bifrontal headaches and left facial paraesthesia	Normal vision, no papilloedema	-	Solid tumor, no calcification or cyst	Subtotal excision leaving behind only a small tumor in the posteroinferior aspect of the third ventricle	Developed a subgaleal CSF leak which required a secondary procedure to close a small dural defect, underwent adjuvant radiotherapy	No
Matthews (10)	1	65	Female	Dementia, two episodes of syncope, mental status changes, no headache, vomiting or papilloedema	No papilloedema	-	Solid tumor, no calcification	Complete excision	Dementia and hydrocephalus remained unchanged after surgery, patient underwent a ventriculoperitoneal shunt and mental status improved steadily	-
Sole-Llenas et al. (20)	1	33	Female	Frontonuchal headache, asthenia, drowsiness, vomiting, occasional psychomotor agitation, attacks of loss of consciousness lasting several min, one episode of urinary incontinence; examination showed left abducent paresis, bilateral exophthalmos, and paresis of gaze, generalized hyper-reflexia, bilateral Babinski sign and vesical incontinence	-	-	Third ventricular tumor with cystic component	Complete excision	Worsening of clinical symptoms 11 months after surgery, CT scan done-intraventricular tumor in anterior third ventricle with extraventricular extension noted	Yes, detected 11 months after surgery, underwent surgery for recurrence
Chin (21)	1	56		Signs and symptoms of raised intracranial pressure	-	-	Mass lesion in the third ventricle on CT scan	-	-	-
Sacher et al. (22)	1	34	Male	Worsening bifrontal headaches	Normal	Normal	CT scan-non-calcified cystic lesion	-	-	-
Fukushima et al. (14)	1	28	Male	Headache, nausea; examination revealed drowsiness, mild optic atrophy and left upper and right lower temporal quadrantanopia	-	-	CT scan-enhancing isodense mass with low density spots in the third ventricle	Partial excision and decompression of optic chiasm	Transient diabetes insipidus, no neurological deficit, visual fields improved	No
Migliore et al. (16)	1	11	Female	Headache, nausea, and vomiting; examination showed bilateral papilloedema and marked bradycardia	-	-	CT scan-spherical hyperdense non-enhancing mass lesion	Complete excision	Uneventful, no cortisone or thyroxine therapy necessary	No
Iwasaki et al. (23)	2	39	Male	Progressive headache; examination showed mild papilloedema	Normal	Normal	Solid mass, no cyst/calcification	Complete excision	Headache resolved, no neurological deficit, postoperative hormonal examinations normal	No
		49	Male	Headache	-	Normal	Lesion in third ventricle, no cystic regions/calcification	Complete excision	Transient mild disorientation, normal endocrinological reports	No
Davies et al. (6)	6	45 (Mean)	3 males 3 females	Headache-4/6, visual symptoms-6/6, hypopituitarism in 3/6, memory disturbances 1/6	Visual symptoms in all patients, homonymous hemianopia in 3/6 patients, bitemporal defect in 1/6, left central scotoma and right temporal defect in 1/6 and transient obscuration in 1/6	Hypopituitarism in 3/6, DI in 1/6	-	Complete excision−1/6, subtotal excision-3/6, partial excision−2/6	Normalization of vision in 3/6, improvement in 2/6 and improvement with later deterioration in 1/6, hypopituitarism in 3/6, DI with adypsia in 2/6, memory loss which recovered in 2/6 and impaired memory in 1/6, obesity in 2/6, all received radiotherapy at some stage	-
Maira et al. (24)	8	33.63 (Mean)	3 males, 5 females	Amenorrhoea 2/8, psychological disturbances 2/8, hydrocephalus 2/8, headache 1/8, hypopituitarism 1/8	-	Panhypopituitarism in 4/8, gonadotropin deficiency in 1/8, hyperprolactinaemia in 2/8	Solid tumor in all patients, no cysts/calcifications	Complete excision−7/8, partial excision in 1/8	No patient received adjuvant radiotherapy, visual functions preserved for all patients, confusion and fluid-electrolyte imbalance in one patient requiring prolonged ICU stay who later recovered but had persistent panhypopituitarism (requiring replacement therapy) and a defective thirst mechanism	1 recurrence
Pascual et al. (25)	1	47	Male	Headache, psychiatric disturbances, memory disturbances	Normal	Normal	Cystic	Cyst decompression and partial excision	Complete recovery, normal neurological examination	No
Madhavan et al. (26)	1	56	Male	Headache, fever, generalized lethargy, drowsiness; on examination there was neck stiffness and papilloedema with left lower limb hypertonia and extensor Babinski response	-	Normal	Solid	Complete excision	Recovered uneventfully after surgery but died before discharge on 12th postoperative day due to acute myocardial infarction	-
Agrawal et al. (27)	1	10	Female	Headache, diminution of vision in the left eye, bilateral papilloedema	Finger counting at 2 feet left eye, right eye normal	-	Cystic, no calcification	Complete excision	Improvement in vision	-
Tayari et al. (15)	1	22	Female	Headache, nausea, and vomiting, one episode of generalized tonic-clonic seizure, fundoscopy showed mild bilateral papilloedema	Normal	-	No calcification	Complete excision	-	No
Pan et al. (2)	17	37.3 ± 14.3 (Mean)	13 males, 4 females	Raised ICP-11/17, cognitive disturbances-8/17, visual deficits-8/17, diabetes insipidus-3/17, somnolence - 3/17	Visual deficits-8/17	Hypoadrenalism in 8/17, hypothyroidism in 6/17, hypogonadism in 14/15 adults, GH deficiency in 9 patients, panhypopituitarism in 3/17	Solid tumor - 6/17, cystic or mixed variety-11/17	Complete excision−13/17, subtotal excision−4/17	One patient died, one patient developed contralateral epidural haematoma, one patient developed postoperative frontal lobe dysfunction, 16 patients underwent resolution of their postoperative hypothalamic symptoms, deterioration of anterior pituitary function seen in all patients (adrenocorticotrophic disturbance in 4/17, thyrotrophic disturbance in 5/17, panhypopituitarism in 3/17, transient DI in 16/17, permanent DI in 5/17)	3/17 patients, all 3 underwent reoperation followed by adjunctive radiotherapy
Yu et al. (12)	24	40.2 (Median), range 15–61 years	M:F ratio-10.5:1	Headache-16/24, visual deficits - 10/24, sexual dysfunction or amenorrhoea-8/24, mental disturbances or drowsiness-8/24, diabetes insipidus-5/24, intracranial hypertension-3/24	Visual deficits-10/24	Sexual dysfunction/ amenorrhoea-8/24	Solid tumor-20/24 cases, cystic tumor-2/24 cases, mixed solid-cystic-2/24 cases	Complete excision−19/24, subtotal excision−5/24	No perioperative mortality, seizure in one patient, mental disturbances, and memory dysfunction in one patient, panhypopituitarism in 8/24, corticotroph insufficiency in 13/24, diabetes insipidus in 15/24	6/24 patients, 1 underwent reoperation, 1 received stereotactic interstitial radiation, 1 patient radiosurgery, 3 patients managed conservatively
Yano et al. (28)	1	52	Male	Headache, fatigue and lethargy, memory disturbances	Left temporal visual field defect	Hypopituitarism with GH deficiency and polyuria	Solid	Complete excision	-	-
Rambarki and Rajesh (11)	2	50	Male	Raised ICP	-	-	Solid-cystic	-	-	-
		27	Male	Raised ICP	-	-	Solid-cystic	-	-	-
Diniz et al. (8)	1	36	Female	Headache, photophobia, phonophobia, vomiting, bilateral papilloedema	-	-	Solid-cystic, no calcification	-	-	-
Kehayov et al. (9)	1	43	Male	Intermittent severe headache and progressive visual loss, obesity	Bitemporal field loss, predominantly for right eye		Solid-cystic	Complete excision	Diabetes insipidus and hypocortisolaemia, complete recovery of vision	No
Guadagno et al. (29)	1	45	Male	Polyuria, polydipsia, polyphagia, severe headache, loss of consciousness	Visual impairment in left eye	Normal	Solid with calcified spots	Near-total excision (>90%)	Death due to meningitis and multi-organ failure	-
Hung et al. (3)	5	46(mean)	1 female, 1 male, the rest not mentioned	Headaches and visual disturbances	Visual disturbances in all five patients	-	Mixed components-2/5, Solid tumors-3/5, no calcification seen in any tumor	Complete excision for 3/5, extent of resection not mentioned for two patients	-	-
Our series	25	35.4	11 males, 14 females	Headache 21/25, vomiting and other features of raised ICP 18/25, memory disturbances 6/25, gait apraxia 5/25, urinary/fecal incontinence 4/25	Visual disturbances in 12/25	Preoperative endocrinological disturbances in 11/25	Cystic-15/25, mixed-8/25, solid-2/25	Complete excision−10/25,near-total excision−9/25,subtotal/partial excision−6/25	DI-7/25 (transient 5/25, permanent 2/25), meningitis 2/25, hydrocephalus requiring VP shunt 4/25, death 3/25	Recurrence 5/25, residual lesion 1/25

## Results

### Demographics

The 25 patients (mean age 35.4 years; age range 6–74 years; male: female ratio = 11:14) with a purely IVCr formed 4.27% (25/585) of all craniopharyngioma patients operated during the same time frame. The six cases of IVCrs presented in the study by Behari et al. have been included in this study (5).

### Clinical Presentation

The clinical signs and symptoms at presentation are summarized in Table 2. Headache (either bifrontal or holocranial) was the most common (84%) symptom at presentation. Other features of raised intracranial pressure like vomiting and transient blurring of vision (due to intermittent abducent nerve palsy brought about by brainstem distortion) were noted in 18 (72%) patients. Three (12%) patients had loss of consciousness attributable to sudden increase in intracranial pressure due to cerebrospinal fluid pathway obstruction at the foramen of Monro.

**Table 2:** Clinical presentation.

	***N* = 25**	**%**
**Clinical symptoms and signs**		
Headache	21	84
Features of raised ICP	18	72
Visual complaints	12	48
Gait apraxia	5	20
Loss of consciousness	3	12
Urinary/fecal incontinence	4	16
Memory disturbances	6	24
Hypothalamic syndrome	3	12
Others	4	16
**Pre op hormonal status**		
Hypothyroidism	6	24
Hypocortisolism	5	20
Diabetes insipidus	4	16
Sexual dysfunction	2	8

Vision was affected in 12 (48%) of the patients. Homonymous hemianopia was seen in two, and bitemporal hemianopia was seen in two patients. Two patients presented with secondary optic atrophy. Six patients had decreased vision with no overt field deficits, and vision could not be evaluated in one patient who presented with altered sensorium. Gait apraxia was present in five (20%) of the patients. Some form of hypopituitarism was present in 11 (44%) of the patients. These endocrinopathies included hypothyroidism, hypocortisolism, diabetes insipidus, irregular menstrual cycle, or loss of libido. Sleep disturbances, changes in appetite or behavior, and cognitive disturbances, as a form of hypothalamic syndrome, was seen in 3/25 (12%) patients.

### Imaging Characteristics

**Figure 3:**
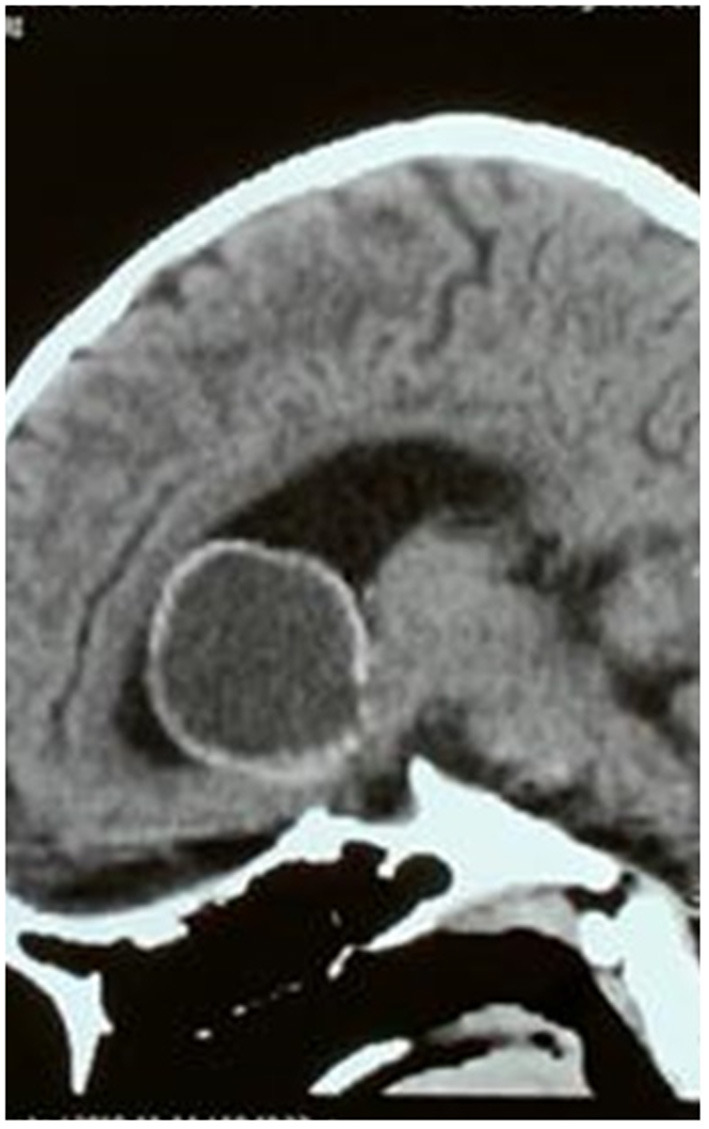
Sagittal section CT scan of a patient with a cystic intraventricular craniopharyngioma showing peripheral calcification, and a bulge in the floor of the third ventricle.

As shown in Table 3, 60% of the tumors were cystic, 8% were solid, and the rest (32%) were of mixed consistency. The cystic component was hyperintense on T2-weighted MRI. Their imaging intensity was distinctly different from the cerebrospinal fluid (CSF) intensity, as noted on FLAIR sequences. Tumors with a predominantly solid component had a more heterogeneous appearance and were most often associated with intramural calcification. In cystic tumors, calcification of a part of the wall was present in six (24%) of the patients (Figure 3). As can be noted in the image shown, in consonance with our selection criteria, the floor of the third ventricle was bulging inferiorly toward the suprasellar cistern in each of the cases due to the mass effect of the tumor but was not breached in any of the cases.

**Table 3:** Imaging characteristics.

	***N* = 25**	**%**
**Type of lesion on imaging**		
Cystic	15	60
Solid-cystic	8	32
Solid	2	8
**Other findings**		
Hydrocephalus	11	44
Calcification	6	24

### Surgical Management

**Figure 4:**
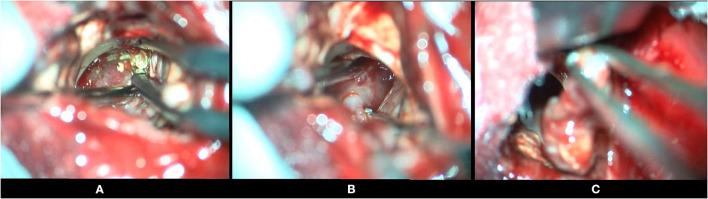
Intraoperative photographs of a patient with an intraventricular craniopharyngioma, undergoing a frontal transcortical-transventricular approach, for tumor excision. **(A)** Tumor being dissected from the walls of the third ventricle (calcifications visualized in the wall of the tumor). **(B)** Tumor being separated from the floor of the third ventricle. **(C)** Solid tumor being removed, after dissection.

While choosing the surgical approach, the factors considered included the age of the patient, the size and site of tumor, the degree of associated hydrocephalus, and the type of visual deterioration. As noted in Table 4, an open craniotomy was used in 18 (72%) patients. Seven patients were operated using the transcortical–transventricular route (Figure 4), of which three underwent an endoscopy-assisted procedure. The interhemispheric-transcallosal approach was used in four patients (of which one was operated using an endoscopy-assisted procedure); the interhemispheric lamina terminalis approach was used in five patients; and, two patients underwent the pterional, trans-sylvian, lamina terminalis approach. In the translateral–third ventricular approaches (transcortical–transventricular and interhemispheric–transcallosal–transventricular; *n* = 11), the foramen of Monro was usually sufficiently dilated to access the third ventricle through the transforaminal route (*n* = 10). The subchoroidal approach was used in one patient in whom the tumor was situated posterior to the foramen of Monro in the body of the third ventricle. Ommaya reservoir placement was done within the cyst in two tumors.

**Table 4:** Surgical details.

**Procedure type(*n*)**	**Approach**	***N***	**%**
Open (18)	Trans-cortical trans-ventricular	7 (3 endoscope-assisted)	16
	Inter-hemispheric trans-callosal	4 (1 endoscope-assisted)	12
	Inter-hemispheric lamina terminalis	5	20
	Pterional trans-sylvian lamina terminalis	2	8
Purely endoscopic (7)		7	28
**Extent of resection**		**N**	**%**
Complete		10	40
Near-total		9	36
Subtotal/partial		6	24

**Figure 5:**
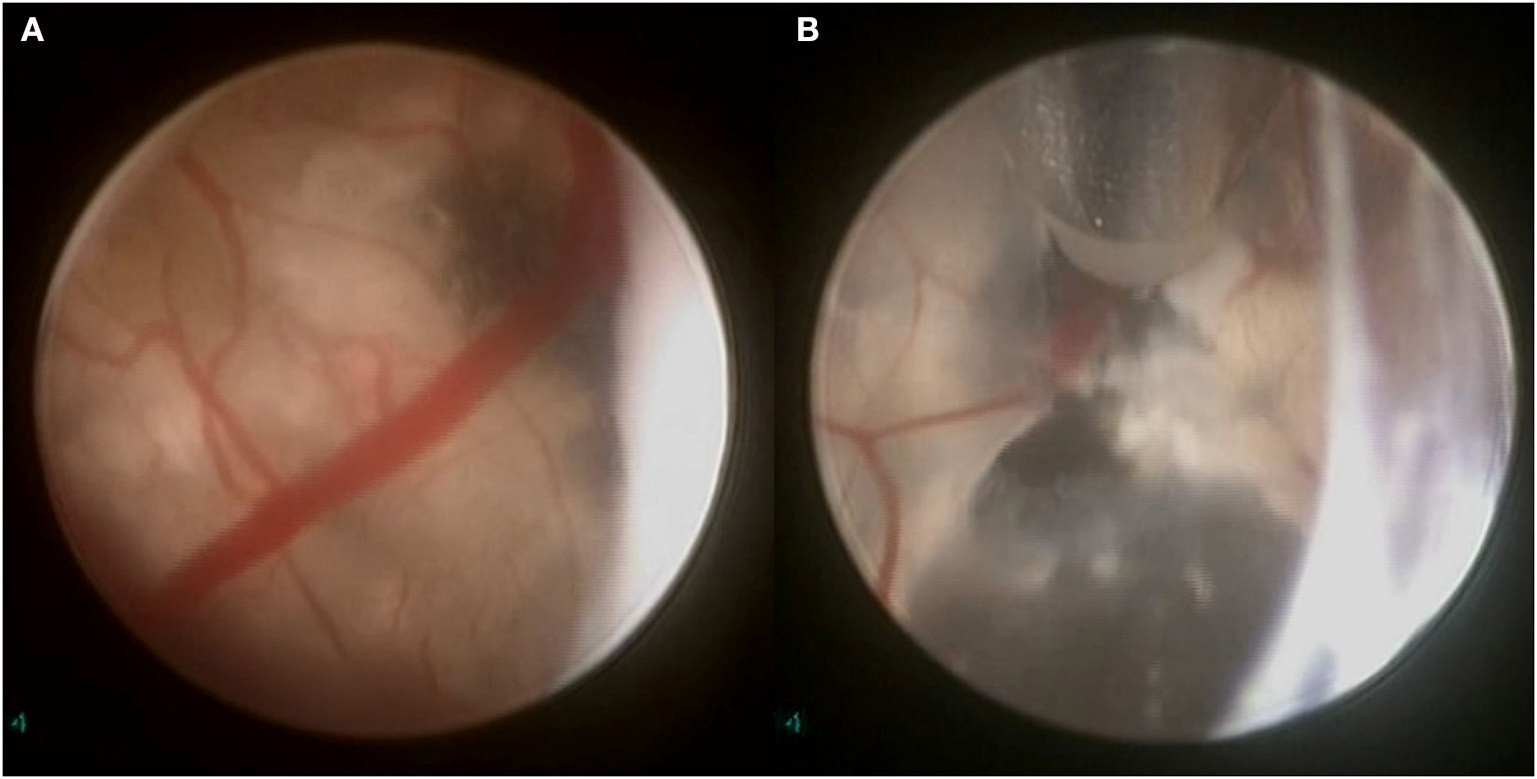
Intraoperative endoscopic view of a cystic intraventricular craniopharyngioma in a 6-year-old child. **(A)** Craniopharyngioma cyst wall (with calcifications) visualized. **(B)** Using bipolar diathermy, a fenestration is made into the cyst wall for draining its contents and insertion of a ventricular catheter connected to an Ommaya reservoir (through which alpha-interferon was administered).

Among those who underwent a purely endoscopic procedure (mainly those with a cystic tumor in whom a transcortical–transventricular approach was adopted; *n* = 7), cyst fenestration (Figure 5) along with wall biopsy was performed in four patients. Endoscopic third ventriculostomy was performed along with cyst fenestration in one patient. Two patients underwent a purely endoscopic procedure, *via* a keyhole craniotomy. One patient underwent a staged procedure with endoscopic cyst fenestration and intracystic interferon (IFN)-alpha therapy, followed by microsurgical excision.

Complete excision of the tumor was possible in 10 (40%) of the patients, while in nine (36%), near-total excision was possible. In the latter cases, the capsule of the tumor, densely adherent to the ventricular wall, was left *in situ*. Only a partial or subtotal removal of the tumor was done in six (24%) patients, with a predominantly cystic tumor having a very thin wall. In the cystic tumors, cholesterol crystals were noted, with the presence of machine-oil colored fluid, after cyst fenestration.

### Post-operative Course

In the immediate postoperative period, diabetes insipidus (DI) was present in seven (28%) of the patients. The DI was transient in five (20%) patients, and two patients (8%) developed permanent DI and requiring long-term vasopressin (desmopressin) supplementation. The postoperative complications are summarized in Table 5. Features of new-onset hypothalamic dysfunction after surgery were seen in one patient. Three patients expired in the series. One of them developed postoperative septicaemia, the second developed central DI with adipsic hypernatraemia, and the third expired due to pulmonary embolism secondary to deep vein thrombosis after second surgery for recurrence, 6 years after primary excision.

**Table 5:** Postoperative complications and follow-up.

	***N***	**%**
**Postoperative complications**		
Transient DI	5	20
Permanent DI	2	8
Hydrocephalus requiring VP shunt	4	16
New-onset hypothyroidism	2	8
New-onset hypocortisolism	2	8
New-onset hypothalamic dysfunction	1	4
Meningitis	2	8
CSF Leak	1	4
Septicaemia	1	4
**Follow up**		
Post-op radiotherapy	8	32
Recurrence	5	20
Residual lesion	1	4

### Follow-Up

**Figure 6:**
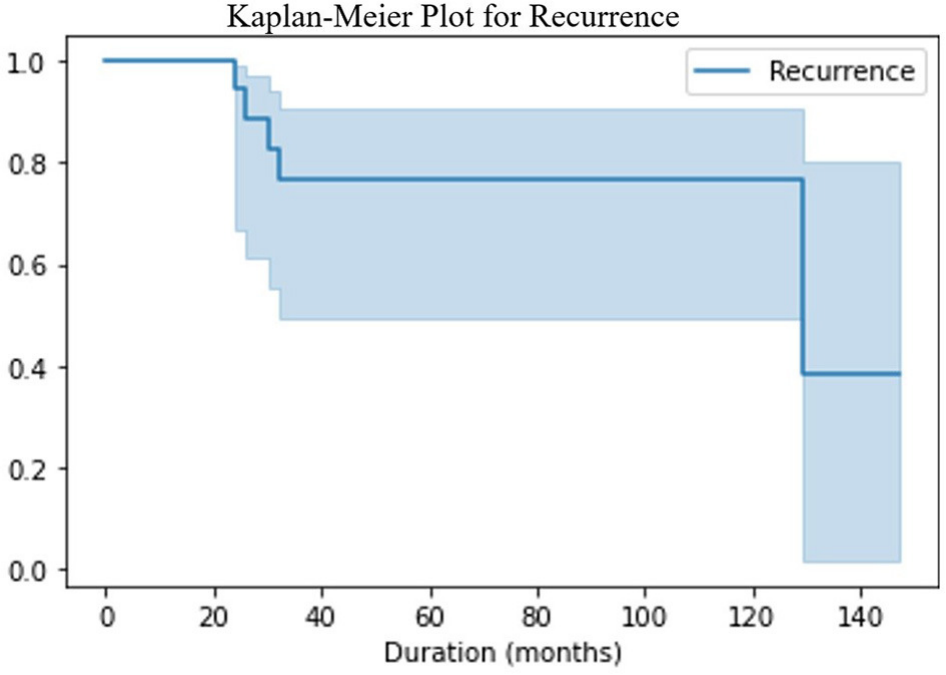
The Kaplan- Meier survival plot showed that majority of the recurrences are detected in the first 36 months, but the risk of recurrence still exists even at 10 years of follow up.

The median follow-up period was 36 months, with a range of 11–147 months. In 17 patients (68%), the follow-up MRI revealed no recurrence; while five patients had a recurrence (Figure 6) and, one patient had a small residual tumor. The patient with the small residue and two others with small cystic recurrences, were observed. Among the other three patients with recurrences, one patient who had a recurrence in the region of the hypothalamus was managed with radiotherapy alone. Another patient underwent re-surgery after a trial of radiotherapy. The last patient with a recurrent lesion, developed a local recurrence which was managed with radiotherapy; and later developed an intraparenchymal ectopic recurrence for which he underwent surgery. The residual cystic collection of two patients was managed conservatively by tapping of the Ommaya reservoir. In all, four out of 11 patients with preoperative hydrocephalus underwent a ventriculoperitoneal shunt (of which, one was before the definitive procedure). One patient with mild transient hydrocephalus was managed conservatively with acetazolamide. The remaining patients did not require any other intervention for CSF diversion.

Radiotherapy of 4,500–5,400 rads was given in eight (32%) patients. Two of them received radiotherapy after developing a recurrence. A maintenance dose of prednisolone (5 and 2.5 mg daily) was given in all patients for a period of 6 weeks, and supplemental thyroxine (0.1 mg/day) was given in the six patients who were preoperatively hypothyroid. On follow up at 6 weeks, a complete hormonal evaluation was done to assess for hypopituitarism, which was accordingly treated with the necessary supplemental therapy. In our series, we observed the development of new-onset hypocortisolism in two (8%) patients, and new-onset hypothyroidism in two (8%) patients, after surgery. Documented postoperative improvement in vision was seen on formal assessment in 6/12 (50%) of the affected patients. Documented formal assessment was not charted for the other five patients but clinical assessment notes show better or stable vision. No further deterioration in vision occurred for any of the patients.

## Discussion

Cushing described craniopharyngiomas as “the most formidable of intracranial tumors” (1). The intraventricular variety is the least common and poses special challenges (5). IVCrs were first reported in 1953 by Dubos et al. and subsequently by Cashion and Young (5, 16, 17, 20, 25). It is widely believed that the adamantinomatous variety originates from epithelial cell rests in the Rathke's pouch and that the papillary variety originates from the squamous metaplasia within the pars tuberalis of the pituitary gland (2). Previously, Atwell had suggested that the pars tuberalis could rarely grow forward or backward, and that it could sometimes extend to the infundibulum or the tuber cinereum; and this was believed to be the cause of the location of these tumours being exclusively in the third ventricle (23, 30). However, it is now known that the pial membrane serves as a barrier preventing the Rathke's pouch cells from coming into direct contact with the developing cerebral vesicle (the precursor of the third ventricular floor and infundibulum). If the development of this pial membrane is delayed, the cells of the Rathke's pouch come into direct contact with the neuroectoderm of the developing cerebral vesicle. These cells, when they grow to form a tumor, give rise to a purely intraventricular craniopharyngioma (5, 8, 24).

The incidence of purely IVCrs among craniopharyngioma patients in our series was 4.27% (25 purely intraventricular craniopharyngiomas out of a total of 585 craniopharyngioma patients who underwent surgery). The incidence reported in other major series is 2.89% (by Yu et al.) and 8.72% (by Pan et al.) (2, 12).

The importance of the location of craniopharyngiomas with respect to the third ventricle has long been recognized, as can be seen from the various classification schemes proposed. Steno (1985) classified craniopharyngiomas into the intrasellar and suprasellar types. Suprasellar types were further classified into three groups (extraventricular, intra–extraventricular, and intraventricular), based on their relationship to the third ventricular floor (31). Yasargil classified craniopharyngiomas into purely intrasellar–infradiaphragmatic; intra and suprasellar, infra and supradiaphragmatic; parachiasmatic, extraventricular; intra- and extraventricular; paraventricular with respect to their location relative to the third ventricle; and purely intraventricular (13, 32). Samii and Tatagiba graded craniopharyngiomas into five types based on the extension of the tumor. Grade I tumors were intrasellar or infradiaphragmatic; grade II tumors were occupying the cistern with/without an intrasellar component; grade III tumors were in the lower half of the ventricle; grade IV tumors were in the upper half of the ventricle; and, grade V tumors were reaching the septum pellucidum or the lateral ventricles (13, 33). Kassam et al. classified craniopharyngiomas after the advent of the endonasal endoscopic route, in terms of their relation to the pituitary stalk, into the preinfundibular, transinfundibular, and retroinfundibular types with a separate isolated intraventricular (type IV) variety, defined as being unsuitable for this approach (13, 34).

However, it was Pascual et al. who described the intricate relationship of the IVCrs relative to the third ventricular floor. The lesions were classified into: the suprasellar tumor pushing the intact third ventricular floor upwards (*pseudo-intraventricular*); suprasellar mass breaking through the third ventricle floor and invading the third ventricle cavity (*secondarily IVC*); intraventricular mass within the third ventricle cavity and floor, the latter being replaced by tumor (*non-strictly IVC or infundibulo-tuberal craniopharyngiomas*); and intraventricular mass completely located within the third ventricle cavity and with the intact floor lying below its inferior surface (*strictly IVC*) (13, 25). We believe that such a topographical classification is necessary to help in choosing the appropriate surgical approach.

IVCrs, though rare, have been reported to occur predominantly in adults (5, 8, 11, 12, 15, 35). The oft cited reason for this predominant occurrence in adult patients is the slow growth of these tumors along with their location in the third ventricle lumen, whereby the symptom onset may be delayed till infiltration into the walls of the ventricle or obstructive hydrocephalus occurs (8, 15). The clinical presentation of IVCrs has been predominantly that of raised intracranial pressure (headache, nausea, vomiting, and papilloedema). In older patients, clinical manifestations of normal-pressure hydrocephalus (cognitive disturbances, gait imbalance, and urinary incontinence) may occur. In our series, we had a mix of pediatric (eight patients) and adult cases (17 patients).

The absence or paucity of the effects of local compression on the optic apparatus or the pituitary stalk/gland (visual symptoms or endocrinological disturbances) is noted in IVCrs (5, 6, 8, 10, 14, 15, 19, 25, 35, 36). In our series, visual symptoms on presentation were seen in 12/25 (48%) of patients. In two of our patients, homonymous hemianopia was observed, which was, likely due to the pressure effect on the optic tract. The two patients who showed bitemporal hemianopia, perhaps manifested this sign due to the bulging of the chiasmatic recess of the third ventricle leading to chiasmatic compression. In some of the other series as well (Yu et al. and Pan et al.) visual deficits were noted in 10/24 and 8/17 patients, respectively (2, 12). Preoperative hypopituitarism was noted in 11 (44%) of our patients, whereas in the series by Yu et al. it was seen in 8/24 (33%) of patients (12). This could be explained by the pressure effect of the tumor on the pituitary stalk or the hypothalamus.

Diabetes insipidus, behavioral changes, autonomic nervous system disturbances such as disturbances in sleep rhythm, appetite, body core temperature, and disturbances in memory, may also be observed (due to hypothalamic dysfunction as a result of the tumor invasion into the lateral wall or floor of the third ventricle) (3, 37–39). Cognitive or psychological disturbances can be due to primary hypothalamic involvement by the tumor, (40) hydrocephalus, or forniceal involvement due to pressure. In our series, we observed memory disturbances, in 6/25 (24%) patients.

**Figure 7:**
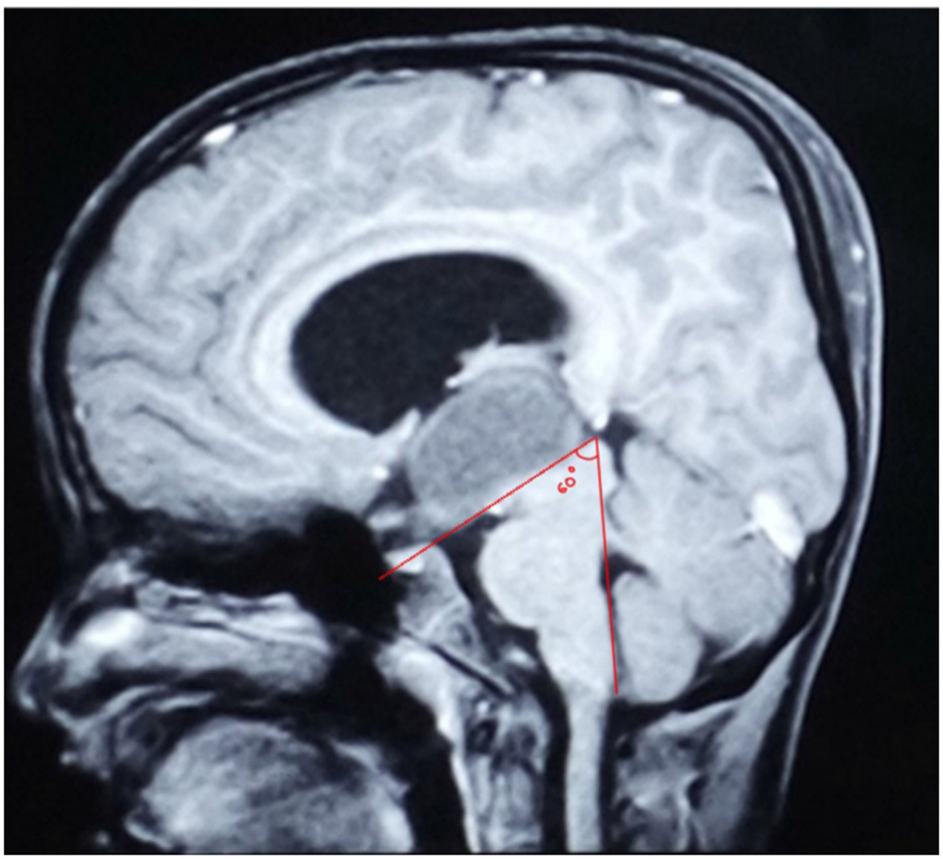
Mid-sagittal MRI image of an 8-year-old boy with a purely intraventricular craniopharyngioma showing the Mamillary Body Angle being 60 degrees.

The integrity of the third ventricular floor has long been recognized as an important radiologic criterion which helps to differentiate between a purely IVCr and one that is primarily suprasellar but with an intraventricular extension (41). Migliore et al. described the other commonly accepted radiologic criteria for purely IVCrs: a patent suprasellar cistern, an intact pituitary stalk, and the absence of sellar abnormalities (8, 16). Pascual et al. proposed that two important signs can significantly correlate with the topographical type of craniopharyngioma-third ventricle relationship. The first of these radiological signs was the identification of the mamillary bodies (MBs) and measurement of the mamillary body angle (MBA, the angle subtended between the intersection of one plane tangential to the base of one of the MBs and the plane tangential to the fourth ventricular floor) on preoperative midsagittal MRI images; and the second sign was the relative position of the hypothalamus with respect to the tumor on coronal preoperative MRI images (42). Chiasmal distortion has been evaluated and found to be a prominent feature that may help in differentiating varieties of IVCrs. It has been found to be stretched upward in *pseudo-intraventricular* tumors, stretched forward in *secondarily IVCs*, compressed forward in *non-strictly IVCs*, and compressed downward in *strictly IVCs* (18, 42, 43). Prieto et al. suggested six characteristics of strictly IVCrs based on the conventional T1 and T2-weighted MR images: a typical rounded shape; the downward deviation of the optic chiasm; a well-observed pituitary stalk; a free chiasmatic cistern; an MBA between 30° and 60° (Figure 7); and the hypothalamus being situated below the lower-third of the tumor (3, 43). These lesions have been described as being predominantly solid tumors in some reports (3, 19) and calcifications are only rarely reported in IVCrs (5, 6, 8, 15). In our series of 25 patients, however, 60% of tumors were predominantly cystic in nature. This could be because most series report cases in adults, whereas our series represents a good mix of children (eight patients) and adults (17 patients).

The differential diagnoses of IVCs includes other lesions of the third ventricle such as colloid cysts, ependymoma, choroid plexus papilloma, hypothalamic glioma, meningioma, germinoma, and lymphoma (3, 5, 8, 10, 15, 19, 23).

On histopathology, purely IVCrs have been classically reported to be of the squamous papillary variety, lacking calcification (3, 10, 12, 23, 26, 27); any associated calcification is in the form of microcalcifications (26). However, Davies et al. Pascual et al. and Yu et al. have found an even distribution between the adamantinomatous and papillary histological varieties of these tumors (6, 12, 25).

The indications for surgical treatment of IVCrs include: a. the need for a tissue diagnosis; and b. the relief of preoperative symptoms with the goal of maximal safe decompression of the lesion. The need for further adjuvant therapy (brachytherapy or radiotherapy, as the case may be) can be decided depending upon the clinical status of the patient and the extent of residual lesion, either in the immediate postoperative period, or on periodic follow-up visits. Stereotactic radiation utilizing gamma knife radiosurgery can also be considered for residual or recurrent cases (3). Proton beam therapy has also shown a great potential (44, 45).

The risks of surgical intervention in the region of the third ventricle poses challenges because of the complexity and critical location of the surrounding structures including the hypothalamus, infundibulum, optic pathway, limbic system, adjacent vascular structures, and fornix (5, 46). Often, the tumor cannot be excised completely, and subtotal excision followed by radiotherapy has been the treatment of choice (6).

Classical approaches used for primary intraventricular tumors have all been described for the management of these lesions (23). These can be broadly divided into the transventricular approaches from the roof of the ventricle, and the lamina terminalis approaches along the floor of the third ventricle. The lamina terminalis corridor can be approached *via* a sub-frontal/anterior interhemispheric route or a pterional trans-sylvian approach. The transventricular corridor (through the lateral ventricle) can be divided into the transcortical–transventricular–transforaminal approach and the interhemispheric–transcallosal–transventricular approach. From the lateral ventricle, entry into the third ventricle can be *via* the transforaminal, subchoroidal, or interforniceal routes (4). The use of neuronavigation as an adjunct, can enhance surgical safety and results (3). Endoscopic or assisted approaches have added a new dimension to the surgical excision of these lesions.

The trans-lamina terminalis approach was first described by (47) in 1936 for the treatment of tumor-related hydrocephalus (24). The lamina terminalis is accessible *via* an anterior interhemispheric approach or a sub-frontal corridor or *via* a more lateral corridor through a pterional craniotomy (4, 9, 18, 24, 48). During the pterional (fronto-temporal) approach, due to the angulation of the surgical trajectory, it may be difficult to visualize the posterior-most region of the third ventricle. Moreover, tumors that are adherent to the lateral wall or floor of the third ventricle may be difficult to excise via this corridor. According to Prieto et al. the strictly intraventricular topography of craniopharyngiomas is associated with a moderate level of severity of adherence (49). The anterior interhemispheric approach on the other hand provides a more direct trajectory and a view of the entire third ventricle via the lamina terminalis. It helps in visualizing the third ventricle posteriorly right up to the aqueduct, and in avoiding hypothalamic injury (2, 5). The lamina terminalis approach was essentially described for retrochiasmal tumors of the extra- as well as intraventricular variety situated predominantly in the midline (50). It is ideal for tumors located predominantly in the inferior part of the third ventricle. It is, however, not suitable for large lesions, for which a transventricular approach is more appropriate (4, 5, 9, 12, 16, 51, 52). The risks associated with the translamina terminalis approach include a retraction injury or perforator damage involving the optic pathway, columns of fornix, supraoptic nuclei, organum vasculosum, and tuber cinereum (5, 9, 53).

Tumors with extensions into the lateral ventricles or those projecting through the foramen of Monro should be considered for an interhemispheric–transcallosal–transventricular approach, or a transcortical–transventricular approach (4, 48). When the lateral ventricle is dilated and the foramen of Monro is enlarged, a natural corridor opens for access to the third ventricular craniopharyngioma. With the transcortical–transventricular approach, the tumor can be dissected from the walls of the third ventricle posteriorly and laterally. A limitation to effectively visualize and dissect the antero-superior aspects and the parts of the lesion adherent to the third ventricular wall ipsilateral to the side of surgical approach arises if the foramen of Monro is not enlarged. In this scenario, a neuroendoscope may help visualize this part of the tumor, and facilitate its excision (54). The other advantages in the use of a neuroendoscope include the confirmation of hemostasis at the end of surgery and the determination of extent of resection on the operating table itself, prior to the performance of a postoperative imaging study. As compared with the transcortical–transventricular–transforaminal route, the interhemispheric–transcallosal–transventricular route may be associated with less tissue damage (and therefore, oedema) and a lower incidence of seizures (4, 5). The interhemispheric–transcallosal approach is advantageous because of the minimal brain retraction and limited division of the corpus callosum needed (5, 16, 28).

**Figure 8:**
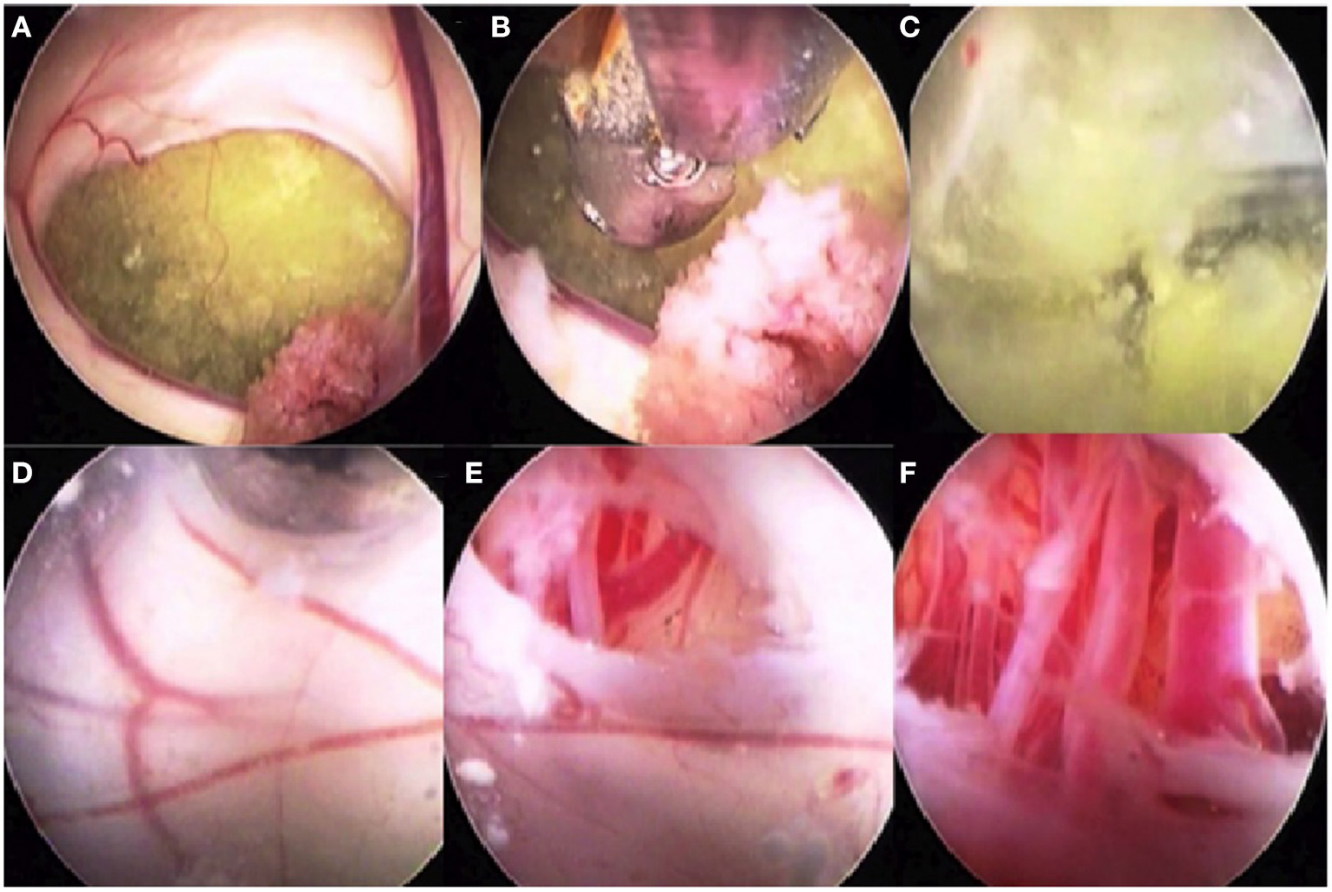
Intraoperative images of a patient with a cystic intraventricular craniopharyngioma undergoing endoscopic cyst fenestration and third ventriculostomy. **(A)** Intra-third ventricular craniopharyngioma cyst seen through foramen of Monro. Choroid plexus is also seen. **(B)** The cyst is being fenestrated using an endoscope through the frontal, transcortical, transforaminal approach. **(C)** The machinery oil fluid of the craniopharyngioma cyst is well seen. **(D)** The inner wall of the craniopharyngioma cyst blended with the third ventricular ependymal floor is seen after drainage of the cyst contents. The cyst wall along with the third ventricular floor is being fenestrated. **(E)** The arterial blood vessels in the interpeduncular cistern are visible. **(F)** The endoscope tip is taken through the inferior cyst wall and the ventricular ependyma to see the blood vessels of the interpeduncular cistern.

Endoscopic fenestration/marsupialisation of cystic craniopharyngiomas in the third ventricle is often done, especially for patients presenting with acutely raised intracranial pressure. The part of the cyst wall emerging at the foramen of Monro, as well as its floor blending with the third ventricular floor, may be fenestrated to gain access to the interpeduncular cistern. This “triple fenestration” (twice of the cyst wall and once of the third ventricular floor) not only helps in decompression of the cyst contents but also in relieving the coexisting hydrocephalus due to the simultaneous performance of the third ventriculostomy (Figure 8). This initial endoscopic treatment can stabilize the neurological condition of the patient and makes the brain less tense for a more invasive microsurgical procedure (55–58). Though a few reports of chemical meningitis are known to occur, it is not a common occurrence (58, 59). An Ommaya reservoir may be placed (60, 61), and instillation of chemotherapy (interferon-alpha) into the cyst may be undertaken for local disease control, if considered appropriate in young children (62, 63). Proponents of this type of therapy favor it, as surgical intervention (either by a trans-cranial or an endonasal trans-sphenoidal approach), has been shown to have a higher rate of postoperative endocrinological disturbances than minimally invasive techniques such as the Ommaya reservoir insertion and cyst aspiration/intracystic therapy (64).

The traditional trans-sphenoidal route has been restricted to intrasellar subdiaphragmatic tumors that extend upwards from an enlarged sella or only partially involve the anteroinferior portion of the third ventricle (as per Grades I and II of Samii's classification) (65). The extended endonasal trans-sphenoidal corridor (extended endonasal approach or EEA) has changed this traditional notion. The latter approach is useful in the management of primarily suprasellar lesions that have an intraventricular extension, especially if the chiasma–pituitary window is not narrow (48, 66–69). The position of the optic chiasm is considered the most important factor in approaching the ventricle via the endonasal route. An anteriorly placed prefixed chiasm provides a good window; however, when this corridor is narrow, techniques such as “pituitary transposition” may help in providing space for the surgical corridor to the subarachnoid cisterns above the optic apparatus and the third ventricle (70). The surgeon must approach the tumor working alternatively from both sides of the stalk. In this manner, these tumors, which often do not cause sellar enlargement, can be successfully removed (65). The benefits of this approach include direct access to ventral midline structures with only minimal handling and/or manipulation of the adjacent neurovascular structures. The major drawback is the lack of a natural corridor in the absence of a suprasellar component of the tumor, and the significant risk of a postoperative CSF leak and its consequences (53, 71).

Perhaps the most important utility of all the classification systems discussed in the context of IVCs, lies in the prediction of the relationship of the third ventricle margins to the tumor (25). In this respect, the classification system proposed by Pascual et al. seems to be the most useful one in planning of surgical approach as it is dependent upon tumor topography and its relationship to the third ventricle. Nine (36%) patients in the current series had adherence of the tumor to the walls of the third ventricle, hence near-total excision was performed to preserve the ventricular walls. Though one can transgress the floor of the third ventricle, as splaying of the floor occurs in retrochiasmal tumors extending into the ventricle (*secondarily IVCr*); in *purely IVCrs*, it is essential that integrity of the third ventricle floor is maintained as there is no splaying of the hypothalamus (48). The exception to the rule may be an extremely thinned-out floor in a cystic tumor where an endoscopic cyst fenestration/removal may be complemented by a third ventriculostomy, as was done in two of our patients. This may rationalize the occasional use of endoscopic endonasal route for purely IVCr (4).

The concept of “maximum safe resection,” traditionally described in neurosurgery for the management of gliomas, has now been adopted in the management of craniopharyngiomas as well, which we know are benign tumors; especially so in children, who have a longer life expectancy and in whom the consequences of hypothalamic dysfunction (and postoperative hormonal disturbances) can significantly impair the quality of life. Most experts nowadays consider subtotal resection to be an acceptable surgical outcome (with the indispensable tool of adjuvant therapy in our armamentarium), provided that most of the tumor is removed, just short of its hypothalamic component or that which is adhered to critical neurovascular structures (72).

Hypothalamic syndrome is an often-described clinical feature in literature, for IVCrs. Children and adolescents usually develop growth failure and disorders of puberty (which may be delayed or precocious). Adults present with cognitive disturbances, disturbances of sleep and appetite, and hormonal disturbances (73). In our series of 25 patients with purely IVCr, we observed preoperative hypothalamic dysfunction in three patients and new-onset postoperative hypothalamic dysfunction in one patient. A total of 4/25 (16%) of our patients with IVCrs, had the hypothalamic syndrome.

It is imperative to pay careful attention to the postoperative fluid balance and body temperature charts as the patients may develop diabetes insipidus and disturbances of body temperature regulation, due to hypothalamic injury (18, 24). Patients may require long-term supplementation of corticosteroid, thyroid, and other pituitary hormones; and diencephalic insufficiency (due to damage to hypothalamic nuclei) may even occur in a delayed fashion even months or years after surgery (18, 24). In our series, two patients (8%) developed permanent diabetes insipidus and five patients (20%) developed transient diabetes insipidus, after surgery.

Adamson et al. have shown that there is a rare chance of recurrence of the squamous papillary variety after complete microsurgical resection. This is because a clear demarcation is usually present between the tumor and the adjacent brain (23, 26, 74). Maira et al. and Pierre-Kahn et al. have shown that a worse postoperative outcome is obtained where there is a disappearance of the third ventricular floor (24, 25, 75). Anatomic preservation of the third ventricular floor and walls and of the infundibulum is truly of significant importance for a good postoperative outcome (2). A good postoperative visual outcome is also generally reported (5, 6).

## Conclusion

IVCrs present predominantly with symptoms of raised intracranial pressure and hydrocephalus. A careful study of the preoperative imaging is necessary to identify them and decide the appropriate surgical approach. The relationship of the tumor to the third ventricle walls and floor, as well as the presence or absence of infiltration of the tumor in the hypothalamus, are important factors in deciding the surgical strategy. Serial clinical and radiological follow-up, with appropriate adjuvant therapy, when necessary, are essential in the management of these patients.

## Data Availability Statement

The original contributions presented in the study are included in the article/Supplementary Material, further inquiries can be directed to the corresponding author/s.

## Ethics Statement

Ethical review and approval was not required for this study on human participants in accordance with local legislation and institutional requirements since this was a retrospective study and there was no deviation from standard of care that was provided to the patients. Written informed consent to participate in this study was provided by the participants' legal guardian/next of kin.

## Author Contributions

KS wrote the manuscript primarily and also contributed to data collection. AK and BT contributed to data collection and formation of the results and assisting with writing the manuscript. VK and CM were part of the surgical team and have contributed with the technical discussion. All authors contributed to the article and approved the submitted version.

## Conflict of Interest

The authors declare that the research was conducted in the absence of any commercial or financial relationships that could be construed as a potential conflict of interest.

## Publisher's Note

All claims expressed in this article are solely those of the authors and do not necessarily represent those of their affiliated organizations, or those of the publisher, the editors and the reviewers. Any product that may be evaluated in this article, or claim that may be made by its manufacturer, is not guaranteed or endorsed by the publisher.

## Abbreviations

IVCr: intraventricular craniopharyngioma
MBs: mamillary bodies
MBA: mamillary body angle
FLAIR: fluid attenuated inversion recovery sequence
MRI: magnetic resonance imaging
DVT: deep venous thrombosis
ICP: intracranial pressure.

## References

[1] Deopujari CE Karmarkar VS Shah N Vashu R Patil R Mohanty C. Combined endoscopic approach in the management of suprasellar craniopharyngioma. Childs Nerv Syst. (2018) 34:871–6. 10.1007/s00381-018-3735-8 29435609

[2] Pan J Qi S Lu Y Fan J Zhang X Zhou J. Intraventricular craniopharyngioma: morphological analysis and outcome evaluation of 17 cases. Acta Neurochir. (2011) 153:773–84. 10.1007/s00701-010-0938-5 21271265

[3] Hung ND Ngan VK Duc NM. Intrinsic third ventricular papillary craniopharyngioma: a report of five cases and literature review. IMCRJ. (2021) 14:83–7. 10.2147/IMCRJ.S295848 33623444 PMC7894859

[4] Cossu G Jouanneau E Cavallo LM Elbabaa SK Giammattei L Starnoni D. Surgical management of craniopharyngiomas in adult patients: a systematic review and consensus statement on behalf of the EANS skull base section. Acta Neurochir. (2020) 162:1159–77. 10.1007/s00701-020-04265-1 32112169

[5] Behari S Banerji D Mishra A Sharma S Sharma S Chhabra DK. Intrinsic third ventricular craniopharyngiomas: report on six cases and a review of the literature. Surg Neurol. (2003) 60:245–52. 10.1016/S0090-3019(03)00132-0 12922045

[6] Davies J King TT Metcal KA Monson JP. Intraventricular craniopharyngioma: a long-term follow-up of six cases. Br J Neurosurg. (1997) 11:533–41. 10.1080/02688699745691 11013625

[7] Graffeo C Perry A Link M Daniels D. Pediatric craniopharyngiomas: a primer for the skull base surgeon. J Neurol Surg B. (2018) 79:065–80. 10.1055/s-0037-1621738 29404243 PMC5796826

[8] Diniz LV Jorge LA Jr Rodrigues LP Vieira Veloso JC Yamashita S. Intraventricular craniopharyngioma: a case report. J Neurol Stroke. (2018) 8:185–8. 10.15406/jnsk.2018.08.00306

[9] Kehayov I Nakov V Kitov B Zhelyazkov H Spiriev T. Interhemispheric transcallosal transforaminal approach and microscopic third ventriculostomy for intraventricular craniopharyngioma associated with asymmetric hydrocephalus: case report and literature review. Folia Med. (2019) 61:143–7. 10.2478/folmed-2018-0049 31237852

[10] Matthews FD. Intraventricular craniopharyngioma. AJNR Am J Neuroradiol. (1983) 4:984–5. 6410885 PMC8333762

[11] Rambarki O Rajesh A. Third ventricular craniopharyngioma. Neurol India. (2016) 64:834–5. 10.4103/0028-3886.185370 27381152

[12] Yu T Sun X Ren X Cui X Wang J Lin S. Intraventricular craniopharyngiomas: surgical management and outcome analyses in 24 cases. World Neurosurg. (2014) 82:1209–15. 10.1016/j.wneu.2014.06.015 24937597

[13] Lubuulwa J Lei T. Pathological and topographical classification of craniopharyngiomas: a literature review. J Neurol Surg Rep. (2016) 77:e121–7. 10.1055/s-0036-1588060 27556005 PMC4993606

[14] Fukushima T Hirakawa K Kimura M Tomonaga M. Intraventricular craniopharyngioma: its characteristics in magnetic resonance imaging and successful total removal. Surg Neurol. (1990) 33:22–7. 10.1016/0090-3019(90)90220-J 2405528

[15] Tayari N Etemadifar M Hekmatnia A Mahzouni P Maghzi AH Rouzbahani R. Intrinsic third ventricular craniopharyngioma: a case report. Int J Prev Med. (2011) 2:178–85. 21811661 PMC3143532

[16] Migliore A Calzolari F Marzola A Ghadirpour R Migliore MM. Intrinsic III ventricle craniopharyngioma. Childs Nerv Syst. (1992) 8:56–8. 10.1007/BF00316565 1576611

[17] Cashion EL Young JM. Intraventricular craniopharyngioma. Report of two cases. J Neurosurg. (1971) 34:84–7. 10.3171/jns.1971.34.1.0084 5539648

[18] King TT. Removal of intraventricular craniopharyngiomas through the lamina terminalis. Acta Neurochir. (1979) 45:277–86. 10.1007/BF01769141 425858

[19] Goldstein SJ Wilson DD Young AB Guidry GJ. Craniopharyngioma intrinsic to the third ventricle. Surg Neurol. (1983) 20:249–53. 10.1016/0090-3019(83)90062-9 6879427

[20] Solé-Llenas J Royo Salvador M Llovet J Sanchez-Larrea R Rovira RR. Craniopharyngioma of the third ventricle. Neurochirurgia. (1983) 26:93–4. 10.1055/s-2008-1053619 6603588

[21] Chin HW. Adult intraventricular craniopharyngioma. Strahlentherapie. (1983) 159:214–6. 6857734

[22] Sacher M Gottesman RI Rothman AS Rosenblum BR Handler MS. Magnetic resonance imaging and computed tomography of an intraventricular craniopharyngioma. Clin Imaging. (1990) 14:116–9. 10.1016/0899-7071(90)90005-v 2372728

[23] Iwasaki K Kondo A Takahashi JB Yamanobe K. Intraventricular craniopharyngioma: report of two cases and review of the literature. Surg Neurol. (1992) 38:294–301. 10.1016/0090-3019(92)90045-O 1440221

[24] Ciric IS. Comments on: Maira G, Anile C, Colosimo C, Cabezas D. Craniopharyngiomas of the third ventricle: trans-lamina terminalis approach. Neurosurgery. (2000) 47:857–65. 10.1097/00006123-200010000-00014 11014425

[25] Pascual JM González-Llanos F Barrios L Roda JM. Intraventricular craniopharyngiomas: topographical classification and surgical approach selection based on an extensive overview. Acta Neurochir. (2004) 146:785–802. 10.1007/s00701-004-0295-3 15254801

[26] Madhavan M. George JP, Jafri JA, Idris Z. Intraventricular squamous papillary craniopharyngioma: report of a case with intraoperative imprint cytology. Acta Cytol. (2005) 49:431–4. 10.1159/000326179 16124175

[27] Agrawal R Misra V Singla M Chauhan SC Singh PA. Intraventricular adamantinomatous craniopharyngioma in a child. Neurol India. (2008) 56:207–9. 10.4103/0028-3886.42008 18688154

[28] Yano S Hide T Shinojima N Ueda Y Kuratsu J. A flexible endoscope-assisted interhemispheric transcallosal approach through the contralateral ventricle for the removal of a third ventricle craniopharyngioma: a technical report. Surg Neurol Int. (2015) 6:S113–6. 10.4103/2152-7806.153653 25883855 PMC4392546

[29] Guadagno E Solari D Pignatiello S Somma T Sgariglia R Ilardi G. A 45-year old man with an intraventricular mass. Brain Pathol. (2020) 30:405–406. 10.1111/bpa.12814 32100434 PMC8018041

[30] Atwell WJ. The development of the hypophysis cerebri in man, with special reference to the pars tuberalis. Am J Anat. (1926) 37:159–93. 10.1002/aja.1000370107

[31] Steno J. Microsurgical topography of craniopharyngiomas. Acta Neurochir Suppl. (1985) 35:94–100. 3867266

[32] Yaşargil MG Curcic M Kis M Siegenthaler G Teddy PJ Roth P. Total removal of craniopharyngiomas. Approaches and long-term results in 144 patients. J Neurosurg. (1990) 73:3–11. 10.3171/jns.1990.73.1.0003 2352020

[33] Samii M Tatagiba M. Surgical management of craniopharyngiomas: a review. Neurol Med Chir. (1997) 37:141–9. 10.2176/nmc.37.141 9059036

[34] Kassam AB Gardner PA Snyderman CH Carrau RL Mintz AH Prevedello DM. Expanded endonasal approach, a fully endoscopic transnasal approach for the resection of midline suprasellar craniopharyngiomas: a new classification based on the infundibulum. J Neurosurg. (2008) 108:715–28. 10.3171/JNS/2008/108/4/0715 18377251

[35] Ikezaki K Fujii K Kishikawa T. Magnetic resonance imaging of an intraventricular craniopharyngioma. Neuroradiology. (1990) 32:247–9. 10.1007/BF00589123 2215911

[36] Zygourakis CC Kaur G Kunwar S McDermott MW Madden M Oh T. Modern treatment of 84 newly diagnosed craniopharyngiomas. J Clin Neurosci. (2014) 21:1558–66. 10.1016/j.jocn.2014.03.005 24908374

[37] Prieto R Pascual JM Hofecker V Winter E Castro-Dufourny I Carrasco R. Craniopharyngioma adherence: a reappraisal of the evidence. Neurosurg Rev. (2020) 43:453–72. 10.1007/s10143-018-1010-9 30043262

[38] Pascual JM Prieto R Carrasco R. Infundibulo-tuberal or not strictly intraventricular craniopharyngioma: evidence for a major topographical category. Acta Neurochir. (2011) 153:2403–25; discussion 2426. 10.1007/s00701-011-1149-4 21918833

[39] Pascual JM Prieto R Rosdolsky M. Craniopharyngiomas primarily affecting the hypothalamus. Handb Clin Neurol. (2021) 181:75–115. 10.1016/B978-0-12-820683-6.00007-5 34238481

[40] Pascual JM Prieto R Castro-Dufourny I Mongardi L Rosdolsky M Strauss S. Craniopharyngiomas primarily involving the hypothalamus: a model of neurosurgical lesions to elucidate the neurobiological basis of psychiatric disorders. World Neurosurg. (2018) 120:e1245–78. 10.1016/j.wneu.2018.09.053 30240857

[41] Schmidt B Gherardi R Poirier J Caron JP. Craniopharyngiome pédiculé du troisième ventricule [Pedicled craniopharyngioma of the 3d ventricle]. Rev Neurol. (1984) 140:281–3. 6718917

[42] Pascual JM Prieto R Carrasco R Barrios L. Displacement of mammillary bodies by craniopharyngiomas involving the third ventricle: surgical-MRI correlation and use in topographical diagnosis. J Neurosurg. (2013) 119:381–405. 10.3171/2013.1.JNS111722 23540270

[43] Prieto R Pascual JM Barrios L. Topographic diagnosis of craniopharyngiomas: the accuracy of MRI findings observed on conventional T1 and T2 images. AJNR Am J Neuroradiol. (2017) 38:2073–80. 10.3174/ajnr.A5361 28935625 PMC7963600

[44] Beltran C Roca M Merchant TE. On the benefits and risks of proton therapy in pediatric craniopharyngioma. Int J Radiat Oncol Biol Phys. (2012) 82:e281–7. 10.1016/j.ijrobp.2011.01.005 21570209 PMC3554244

[45] Tonse R Noufal MP Deopujari CE Jalali R. India's first proton beam therapy pediatric patient. Neurol India. (2020) 68:189–91. 10.4103/0028-3886.279686 32129277

[46] Cavallo LM Di Somma A de Notaris M Prats-Galino A Aydin S Catapano G. Extended endoscopic endonasal approach to the third ventricle: multimodal anatomical study with surgical implications. World Neurosurg. (2015) 84:267–78. 10.1016/j.wneu.2015.03.007 25827043

[47] Maira G Anile C Colosimo C Cabezas D. Craniopharyngiomas of the third ventricle: trans-lamina terminalis approach. Neurosurgery. (2000) 47:857–63. 11014425 10.1097/00006123-200010000-00014

[48] Almeida JP Workewych A Takami H Velasquez C Oswari S Asha M. Surgical anatomy applied to the resection of craniopharyngiomas: anatomic compartments and surgical classifications. World Neurosurg. (2020) 142:611–25. 10.1016/j.wneu.2020.05.171 32987617

[49] Prieto R Pascual JM Rosdolsky M Castro-Dufourny I Carrasco R Strauss S. Craniopharyngioma adherence: a comprehensive topographical categorization and outcome-related risk stratification model based on the methodical examination of 500 tumors. Neurosurg Focus. (2016) 41:E13. 10.3171/2016.9.FOCUS16304 27903121

[50] Bhagwati SN Deopujari CE Parulekar GD. Lamina terminalis approach for retrochiasmal craniopharyngiomas. Childs Nerv Syst. (1990) 6:425–9. 10.1007/BF00302085 2095298

[51] Konovalov AN. Third ventricle craniopharyngiomas. World Neurosurg. (2014) 82:1023–5. 10.1016/j.wneu.2014.08.017 25153290

[52] Coppens JR Couldwell WT. Staged use of the transsphenoidal approach to resect superior third ventricular craniopharyngiomas. Minim Invasive Neurosurg. (2010) 53:40–3. 10.1055/s-0029-1246160 20376745

[53] Forbes JA Ordóñez-Rubiano EG Tomasiewicz HC Banu MA Younus I Dobri GA. Endonasal endoscopic transsphenoidal resection of intrinsic third ventricular craniopharyngioma: surgical results. J Neurosurg. (2018) 1:1–11. 10.3171/2018.5.JNS18198 30497140

[54] Chamoun R Couldwell WT. Transcortical-transforaminal microscopic approach for purely intraventricular craniopharyngioma. Neurosurg Focus. (2013) 34(1 Suppl):Video 4. 10.3171/2013.V1.FOCUS12347 23282157

[55] Adeolu AA Osazuwa UA Oremakinde AA Oyemolade TA Shokunbi MT. Combined microsurgical extra-axial and transcortical transventricular endoscopic excision of parasellar tumors with ventricular extension. Ann Afr Med. (2015) 14:155–8. 10.4103/1596-3519.149891 26021397

[56] Abdullah J Caemaert J. Endoscopic management of craniopharyngiomas: a review of 3 cases. Minim Invasive Neurosurg. (1995) 38:79–84. 10.1055/s-2008-1053462 7583365

[57] Cappabianca P Cinalli G Gangemi M Brunori A Cavallo LM de Divitiis E. Application of neuroendoscopy to intraventricular lesions. Neurosurgery. (2008) 62(Suppl 2):575–97; discussion 597–8. 10.1227/01.neu.0000316262.74843.dd 18596446

[58] Lauretti L Legninda Sop FY Pallini R Fernandez E D'Alessandris QG. Neuroendoscopic treatment of cystic craniopharyngiomas: a case series with systematic review of the literature. World Neurosurg. (2018) 110:e367–73. 10.1016/j.wneu.2017.11.004 29133004

[59] Chen JX Alkire BC Lam AC Curry WT Holbrook EH. Aseptic meningitis with craniopharyngioma resection: consideration after endoscopic surgery. J Neurol Surg Rep. (2016) 77:e151–5. 10.1055/s-0036-1593470 27722072 PMC5053819

[60] Frio F Solari D Cavallo LM Cappabianca P Raverot G Jouanneau E. Ommaya reservoir system for the treatment of cystic craniopharyngiomas: surgical results in a series of 11 adult patients and review of the literature. World Neurosurg. (2019) 132:e869–77. 10.1016/j.wneu.2019.07.217 31400528

[61] Cavallo LM Solari D Somma T Baiano C D'Avella E Cappabianca P. How to manage recurrent craniopharyngiomas. In: Jouanneau E. Raverot G., editors. Adult Craniopharyngiomas. Switzerland, AG: Springer Nature (2020). p. 131–43. 10.1007/978-3-030-41176-3_8

[62] Dastoli PA Nicácio JM Silva NS Capellano AM Toledo SR Ierardi D. Cystic craniopharyngioma: intratumoral chemotherapy with alpha interferon. Arq Neuropsiquiatr. (2011) 69:50–5. 10.1590/S0004-282X2011000100011 21359423

[63] Bartels U Laperriere N Bouffet E Drake J. Intracystic therapies for cystic craniopharyngioma in childhood. Front Endocrinol. (2012) 3:39. 10.3389/fendo.2012.00039 22654864 PMC3356106

[64] Sweeney KJ Mottolese C Villanueva C Beuriat PA Szathmari A Di Rocco F. Adult versus paediatric craniopharyngiomas: which differences? In: Jouanneau E. Raverot G., editors. Adult Craniopharyngiomas. Switzerland, AG: Springer Nature (2020). p. 187–207. 10.1007/978-3-030-41176-3_11

[65] de Divitiis E Cappabianca P Cavallo LM Esposito F de Divitiis O Messina A. Extended endoscopic transsphenoidal approach for extrasellar craniopharyngiomas. Neurosurgery. (2007) 61(5 Suppl 2):219–27; discussion 228. 10.1227/01.neu.0000303220.55393.73 18091236

[66] Algattas H Setty P Goldschmidt E Wang EW Tyler-Kabara EC Snyderman CH. Endoscopic endonasal approach for craniopharyngiomas with intraventricular extension: case series, long-term outcomes, and review. World Neurosurg. (2020) 144:e447–59. 10.1016/j.wneu.2020.08.184 32890848

[67] Koutourousiou M Gardner PA Fernandez-Miranda JC Tyler-Kabara EC Wang EW Snyderman CH. Endoscopic endonasal surgery for craniopharyngiomas: surgical outcome in 64 patients. J Neurosurg. (2013) 119:1194–207. 10.3171/2013.6.JNS122259 23909243

[68] Mou J Wang X Huo G Ruan L Jin K Tan S. Endoscopic endonasal surgery for craniopharyngiomas: a series of 60 patients. World Neurosurg. (2019) 1878–8750. 10.1016/j.wneu.2018.12.110 30610976

[69] Patel VS Thamboo A Quon J Nayak JV Hwang PH Edwards M. Outcomes after endoscopic endonasal resection of craniopharyngiomas in the pediatric population. World Neurosurg. (2017) 108:6–14. 10.1016/j.wneu.2017.08.058 28838874

[70] Nishioka H Fukuhara N Yamaguchi-Okada M Yamada S. Endoscopic endonasal surgery for purely intrathird ventricle craniopharyngioma. World Neurosurg. (2016) 91:266–71. 10.1016/j.wneu.2016.04.042 27108029

[71] Cavallo LM Solari D Esposito F Cappabianca P. The endoscopic endonasal approach for the management of craniopharyngiomas involving the third ventricle. Neurosurg Rev. (2013) 36:27–37; discussion 38. 10.1007/s10143-012-0403-4 22791074

[72] Sarkar S Chacko SR Korula S Simon A Mathai S Chacko G. Long-term outcomes following maximal safe resection in a contemporary series of childhood craniopharyngiomas. Acta Neurochir. (2021) 163:499–509. 10.1007/s00701-020-04591-4 33078364

[73] Shaikh MG. Hypothalamic dysfunction (hypothalamic syndromes). Oxford Textbook of Endocrinology and Diabetes. (2021) Oxford University Press. Available online at: https://oxfordmedicine.com/view/10.1093/med/9780199235292.001.1/med-9780199235292-chapter-241 (accessed September 16 2021)

[74] Adamson TE Wiestler OD Kleihues P Yaşargil MG. Correlation of clinical and pathological features in surgically treated craniopharyngiomas. J Neurosurg. (1990) 73:12–7. 10.3171/jns.1990.73.1.0012 2352012

[75] Pierre-Kahn A Recassens C Pinto G Thalassinos C Chokron S Soubervielle JC. Social and psycho-intellectual outcome following radical removal of craniopharyngiomas in childhood. A prospective series. Childs Nerv Syst. (2005) 21:817–24. 10.1007/s00381-005-1205-6 16049724

